# Synchronized activation of striatal direct and indirect pathways underlies the behavior in unilateral dopamine‐depleted mice

**DOI:** 10.1111/ejn.14344

**Published:** 2019-01-30

**Authors:** Omar Jáidar, Luis Carrillo‐Reid, Yoko Nakano, Violeta Gisselle Lopez‐Huerta, Arturo Hernandez‐Cruz, José Bargas, Marianela Garcia‐Munoz, Gordon William Arbuthnott

**Affiliations:** ^1^ Okinawa Institute of Science and Technology Graduate University Okinawa Japan

**Keywords:** assemblies, basal ganglia, calcium imaging, optogenetics, Parkinson's disease

## Abstract

For more than three decades it has been known, that striatal neurons become hyperactive after the loss of dopamine input, but the involvement of dopamine (DA) D1‐ or D2‐receptor‐expressing neurons has only been demonstrated indirectly. By recording neuronal activity using fluorescent calcium indicators in D1 or D2 eGFP‐expressing mice, we showed that following dopamine depletion, both types of striatal output neurons are involved in the large increase in neuronal activity generating a characteristic cell assembly of particular neurons that dominate the pattern. When we expressed channelrhodopsin in all the output neurons, light activation in freely moving animals, caused turning like that following dopamine loss. However, if the light stimulation was patterned in pulses the animals circled in the other direction. To explore the neuronal participation during this stimulation we infected normal mice with channelrhodopsin and calcium indicator in striatal output neurons. In slices made from these animals, continuous light stimulation for 15 s induced many cells to be active together and a particular dominant group of neurons, whereas light in patterned pulses activated fewer cells in more variable groups. These results suggest that the simultaneous activity of a large dominant group of striatal output neurons is intimately associated with parkinsonian symptoms.

**Abbreviations**
6‐OHDA: 6‐hydroxydopamine
AAV: adeno‐associated viruses
ANOVA: Analysis of variance
BAC: bacterial artificial chromosome
CCD: cooled charge‐coupled device
ChR2: channelrhodopsin variant 2
D1: DA receptor 1
D2: DA receptor 2
DA: Dopamine
EF1a: elongation factor‐1 alpha
eGFP: enhanced fluorescent protein
Fluo‐#: calcium indicator
hSyn: human promoter of synapsin 1
LED: light‐emitting diode
PBS: phosphate‐buffered saline
PD: Parkinson disease
RCaMP1h: red calcium indicator
SPNs: spiny projection neurons

## 1 INTRODUCTION

Since pioneering work in the 1970s we know that unilateral destruction of dopamine (DA) neurons in the rodent mesencephalon induces unilateral hypokinesia, deviated posture, and spontaneous circling or turning behavior toward the lesion side (Ungerstedt & Arbuthnott, 1970). This experimental model of Parkinson's disease (PD) was initially useful in pharmacological studies and it is still useful in the characterization of function of basal ganglia outputs (Greco et al., 2008). However, one of the most striking initial findings using this model is that unilateral destruction of striatal DA input is accompanied by an increase in activity of striatal neuronal activity on the ipsilateral side (Arbuthnott, 1974; Hull, Levine, Buchwald, Heller, & Browning, 1974; Ohye, Bouchard, Boucher, & Poirier, 1970; Schultz, 1982; Schultz & Ungerstedt, 1978).

It is characteristic of striatal projection neurons (SPNs), in anaesthetized animals, that in spite of a correlation between cortical rhythmic activity and membrane potential fluctuations (up and down states) they do not fire rhythmically or synchronously in the presence of DA. However, in the absence of DA, the cortically induced rhythmicity is dramatically increased from less than 1 burst/min to more than 2.5 bursts/min as seen in anesthetized (Arbuthnott, 1974; Nisenbaum, Stricker, Zigmond, & Berger, 1986; Schultz & Ungerstedt, 1978) and awake animals (Chen, Morales, Woodward, Hoffer, & Janak, 2001; Kish, Palmer, & Gerhardt, 1999; Ohye et al., 1970) as well as in vitro (Calabresi, Mercuri, Sancesario, & Bernardi, 1993; Galarraga, Bargas, Martinez‐Fong, & Aceves, 1987). Rhythmic bursting of SPN output targets in the absence DA is a consistent finding (Bergman, Wichmann, Karmon, & DeLong, 1994; MacLeod, Ryman, & Arbuthnott, 1990; Murer, Riquelme, Tseng, & Pazo, 1997; Murer, Tseng, Kasanetz, Belluscio, & Riquelme, 2002; Ni, Bouali‐Benazzouz, Gao, Benabid, & Benazzouz, 2000; Nini, Feingold, Slovin, & Bergman, 1995; Pan & Walters, 1988; Rohlfs et al., 1997; Sanderson, Mavoungou, & Albe‐Fessard, 1986; Wichmann et al., 1999).

Concurrent pharmacological blockade of DA receptor 1 (D1) and DA receptor 2 (D2) SPNs produces an akinesia (often mischaracterized as catalepsy) similar to a DA lesion, but specific blockade of D1 or D2 SPNs indicates that synchronous activity is affected in greater extent by D1 compared to D2 receptor antagonists (Burkhardt, Jin, & Costa, 2009).

In order to understand the role of DA in specific subpopulations of SPNs we used calcium imaging of individual D1‐ or D2‐expressing enhanced eGFP to visualize their activity and study their characteristic patterns of activation in cell assemblies. Moreover, we used calcium imaging and optogenetics to illustrate the specific contribution of both D1 and D2 microcircuits in the generation of the pathological circling behavior characteristic of the animal model of PD.

## 2 MATERIAL AND METHODS

### 2.1 Ethical standards

We used a total of 64 of 78 (14 discarded as failures in procedure or stereotaxic coordinates) Swiss Webster bacterial artificial chromosome (BAC) transgenic mice D1‐ enhanced fluorescent protein (eGFP) and D2‐eGFP (postnatal 21–25 days, males) or C57BL/6J control mice were used. Animals were bred in the university's animal facilities. In general, we adjust the number of animals in our experiments according to the mortality rates after surgeries, the success rate of stereotaxic placement of viral injections and the general success of the experiments considering the expertise of trainees. Our experiments complied with the Guide for the care and use of laboratory animals of the US National Institutes of Health; the Society for Neuroscience Policy on the Use of Animals in Neuroscience Research and the Guiding Policies and Principles for Experimental Procedures endorsed by the government of Japan and supervised by the local Animal Care and Use Committee, approved protocol number 2018‐212‐2.

### 2.2 Stereotaxic surgeries

Surgeries were performed under aseptic conditions in a small animal stereotaxic instrument (Leica Angle Two, Leica Biosystems, Wetzlar, Germany). Isoflorane inhalation (IsoFlo, Abbot, 1.0%–1.5% in medical O_2_) was used as anesthetic and the respiratory rate, heart rate, and hindpaw withdrawal reflexes were monitored throughout the procedure to maintain an adequate anesthetic level as stably as possible. Animals were fixed with atraumatic earbars, the cornea protected with mycochlorin ointment 2% and the skin rubbed with anesthetic gel (10% ketoprofen and lidocaine). Post‐operative care measures to control hypothermia, dehydration and pain included keeping mice warm until they came out of anesthesia (<15 min) by placing them on a disposable heating pad, administering sterile saline (0.5 ml, s.c.) immediately after surgery and leaving available, during the first 72 hr after surgery, a can of diet gel with carprofen (e.g., MediGel CPF, Portland, ME, USA). One‐to‐two weeks after surgery, animals were tested or the brain extracted.

For the intracerebral unilateral administration of the neurotoxin 6‐hydroxydopamine (6‐OHDA, Sigma), mice were premedicated 1 hr before surgery with desipramine HCl (25 mg/kg base, in 0.9% sterile saline, subcutaneous). The 6‐OHDA (0.5 μl of 4 mg/ml in 0.9% NaCl and 0.5% ascorbate) was slowly injected (0.01 μl/min) at stereotaxic coordinates (Franklin & Paxinos, 2008) aimed to the left substantia nigra pars compacta (bregma: AP, −2.3 mm; L, 0.7 mm and from dura: V, −4.3 mm). These animals were challenged with apomorphine (Sigma, 0.01 mg/kg, subcutaneous) 1 week after surgery and turning behavior was automatically evaluated by protocols written under Ethovision XT 11 animal tracking system (Noldus, Wageningen, The Netherlands). Behavioral measurements were counted for 5 min after being placed in the recording environment. Only animals showing robust turning (>5 turns/min) were considered to have adequate DA depletion.

To induce viral infection in SPNs, adeno‐associated viruses (AAV) were intracerebrally delivered D1‐eGFP and D2‐eGFP BAC mice. The AAV10 under the human origin elongation factor‐1 alpha (EF1a) promoter was used to drive expression of channelrhodopsin variant 2 (ChR2) and the fluorescent protein 2mCherry. The AAV1 under the human promoter of synapsin 1 (hSyn) was used for the expression of the genetically engineered red calcium indicator (RCaMP1h, Penn Vector Core, AV‐1‐PV3010). Either a single AAV (0.3 μl) or a combination of both (0.15 μl each) was unilaterally injected in the left dorsal striatum of D1‐eGFP and D2‐eGFP BAC mice (AP, 0.98 mm; L, 1.89 mm; V −3.45 mm) (Franklin & Paxinos, 2008).

Animals that received AAV1 RCaMP1h were left for at least 2 weeks to recover and allow viral expression before in vitro recording experiments. Animals that received AAV‐10 ChR2‐2mCherry injection had a longer surgery since a 3 mm stainless steel guide cannula (25 gauge) was inserted above the injection site and fixed to the skull with a small amount of dental cement (Super‐Bond C&B, Sun Medical) followed by a 5‐mm‐long optic fiber (diameter: 260 μm, length 5.45 mm, Teleopto, Nagoya, Japan) placement into the injection site. A device containing the optic fiber, the high‐intensity light‐emitting diode (LED; 470 nm) and a connector to plug the wireless receiver was also fixed to the skull with more dental cement.

### 2.3 Behavioral observations

To allow viral expression, animals were housed for at least 2 weeks before any recording or behavioral observation was performed. A couple of days before the experiments, animals were first trained for 20 min to carry a mock receiver (12 × 18 × 7 mm, 2 g) plugged to the wireless stimulation system in their home cage. On the experiment day, the infrared receiver (Teleopto, Nagoya, Japan) with the same dimensions and weight, replaced the mock receiver and animals were placed in a rectangular (45 × 20 × 15 cm) or circular (15 cm diameter × 20 cm high) testing chamber.

The stimulation device (Teleopto, Nagoya, Japan) triggered a LED of 470 nm (blue light) with intensity at the tip of 1.0 mW. Behavioral procedures consisted of a period of habituation (≈5 min) followed by a sequence of three periods repeated 10 times: 20 s pre‐stimulation, 15 s of light stimulation either delivered continuously or in patterned pulses (6 ms pulses at 14 Hz) and a 30–60 s post‐stimulation period. Turning behavior was measured and evaluated automatically by protocols written under Ethovision XT 11 animal tracking system (Noldus, Wageningen, The Netherlands).

### 2.4 Corticostriatal slice preparation

Mice were anesthetized with isoflorane inhalation and perfused transcardially using cold saline containing (in mM): 124 choline chloride, 2.5 KCL, 1.3 MgCl_2_, 26 NaHCO_3_, 1.2 NaH_2_PO_4_–H_2_O, 1 CaCl_2_ and 10 glucose saturated with 95% O_2_ and 5% CO_2_, pH = 7.4, 298 mOsm/L. Sagittal corticostriatal slices (200–250 μm) were cut (Leica VT1200S, Nussloch, Germany) and transferred to regular artificial cerebral spinal fluid containing the following in mM: 123 NaCl, 3.5 KCl, 1 MgCl_2_, 1 CaCl_2_, 26 NaHCO_3_, and 11 glucose, saturated with 95% O_2_ and 5% CO_2_, where they remained at room temperature (21–25°C) for at least 1 hr before recording.

### 2.5 In vitro recordings of neuronal activity

For recordings of neuronal calcium activity Fluo‐4 or RCaMP1h, corticostriatal slices (210–250 μm) of D1‐eGFP or D2‐eGFP BAC mice were visualized using an upright microscope (Olympus BX51W1F, Japan, 100W halogen lamp) fitted with brightfield (UM Plan FL 10×) and water immersion (10× Olympus) objective. The microscope was also equipped with a cooled charge‐coupled device (CCD) camera (PCO.EDGE, Kelheim, Germany) with a field view of 800 × 600 μm; acquisition of images was set to 5–6 images/s. At the beginning of each experiment coordinates and landmarks of the displayed image were stored for reference and subsequent analysis. Experimental data were recorded using the CCD camera with acquisition protocols written in camware software. Short movies (180 s, 50–100 ms exposure, 250–500 ms/frames) were taken at time intervals of 5–20 min during 1–2 hr). At the end of the experiment potassium depolarization (mM): 50 KCl, 120 NaCl, 10 HEPES‐Na, and 2 CaCl_2_ pH7.4, was applied for 5 s to validate slice integrity, slices with small numbers of responsive cells (<10) were discarded from the analysis.

When a cell‐permeant calcium indicator was used, slices were incubated in the dark at 38°C for 20–30 min with Fluo‐4 (Fluo‐5AM in few occasions) in 0.1% dimethyl sulfoxide diluted to a final concentration of 10 μM, pH 7.4 (95%O_2_ and 5%CO_2_). Once loaded with the calcium indicator, slices were transferred back to the microscope's recording chamber perfused with artificial cerebrospinal fluid.

The pair ChR2 and RCaMP provided independent addressable spectral channels. For neuronal activation with ChR2 and imaging via the genetically encoded red calcium indicator RCaMP1h and RCaMP1 h activation was achieved using excitation pulses at 580 nm delivered by a xenon‐arc lamp of (Lambda LS Sutter Instruments) and a bandpass filter (FB580‐10, ThorLabs, Newton, NJ, USA). A combined non‐overlapping calcium imaging/optogenetics was possible since light of 580 nm—the peak activation for RCaMP1h—does not activate ChR2 (Glock, Nagpal, & Gottschalk, 2015). The emitted RCaMP1h fluorescence was passed through a long pass filter (550 nm, FEL0550, ThorLabs, Newton, NJ, USA) (Akerboom et al., 2013). Optogenetic stimulation (488 nm) was achieved using a fiber‐coupled LED light source (DC2100, OGKR2 Thorlabs. Newton, NJ, USA). The same optogenetic stimulation parameters (continuous or in 14 Hz pulses) were used in slices and behaving animals.

For depolarization block assessment electrical neuronal activity of ChR2‐/RCaMp1h‐ expressing neurons was recorded during 30 s of continuous light stimulation emitted from the fiber‐coupled LED with the same intensity at the tip than the in vivo configuration (1 mW). Electrical neuronal activity was recorded using borosilicate glass micropipettes (Harvard Apparatus 30‐0057, Holliston, MA, USA) heat polished to obtain direct current resistances of ∼4–6 MΩ. Micropipettes were filled with an internal solution containing in mM: 115 KH_2_PO_4_, 2 MgCl_2_, 10 HEPES, 0.5 EGTA, 0.2 Na_2_ATP, and 0.2 Na_3_GTP. The recordings were made with a microelectrode amplifier with bridge and voltage clamp modes of operation (BVC‐700A, Dagan Co, Minneapolis, MN, USA). Conventional characterization of neurons was made in voltage and current clamp configurations. Access resistances were continuously monitored to be less than 20 MΩ, experiments with changes over 20% were interrupted and terminated. Software designed in LabVIEW environment (National Instruments, Austin, Texas, USA) was used for data acquisition and we performed analysis with Origin (version 8.6, Microcal, Northampton, MA, USA).

During recordings of calcium or electrical activity, slices received a constant flow of artificial cerebral spinal fluid of 2.5 ml/min.

### 2.6 Processing of neuronal calcium images

For this processing we used the methods previously described (Carrillo‐Reid et al., 2008; Jaidar et al., 2010). Briefly, Image J (v.1.45s, National Institutes of Health, WA), MATLAB (Math‐Works, Inc., Natick, MA, USA) and programs written in IDL (Exelis‐Harris software version 8.2) (Cossart, Aronov, & Yuste, 2003; Mao, Hamzei‐Sichani, Aronov, Froemke, & Yuste, 2001; Schwartz et al., 1998) were used to analyze the movies acquired during the recordings (see above). For each movie frame neurons were identified, their contours defined and the mean fluorescence measured as a function of time. Changes in fluorescence were computed as (Fi–Fo)/Fo, where Fi = fluorescence intensity and Fo = resting fluorescence, that is, median fluorescence of the first four frames of the movie. The calcium signals elicited by action potentials were detected based on a threshold value given by their first derivative over time (2.5× standard deviation of the noise). Images were inspected one by one to remove artifacts and slow calcium transients, most likely from glial cells (Carrillo‐Reid et al., 2008; Cossart et al., 2003; Jaidar et al., 2010; Sasaki, Matsuki, & Ikegaya, 2007). As previously described (Miller, Ayzenshtat, Carrillo‐Reid, & Yuste, 2014) to determine whether peaks of synchrony were significant (number of coactive neurons per peak), binary activity from recorded calcium imaging (calcium spikes) were shuffled 1,000 times by randomly transposing intervals of activity within each cell. We then set a threshold corresponding to a significance level of *p *<* *0.05 for peak detection.

### 2.7 Visualization of network states

The vectorization of all recorded activity was performed to express network activity as a function of time. Active and inactive elements were therefore represented within a 250 ms time bin. With this analysis, association of bursting activity of synchronized cells occurring at a particular time can be easily identified (Carrillo‐Reid et al., 2008, 2009; Sasaki, Kimura, Tsukamoto, Matsuki, & Ikegaya, 2006; Sasaki et al., 2007). The inner product of all possible vector pairs (equivalent to the cosine of the angle between the vectors) was used to construct a similarity matrix of all neurons firing in synchrony. This process graphically reveals whether vectors have same or similar components (Sasaki et al., 2006, 2007). To follow network dynamics within the neuronal microcircuits and determine how neuronal activity travels among the different network states in the field of observation, we analyzed the data using multidimensional reduction techniques previously described (Carrillo‐Reid et al., 2008, 2009; Jaidar et al., 2010). Briefly, (a) To reduce the dimensionality of population vectors representing network states, we applied locally linear embedding (LLE), a technique that preserves the structure of nonlinear multidimensional data (Brown, Joseph, & Stopfer, 2005; Carrillo‐Reid et al., 2008; Roweis & Saul, 2000). (b) To choose the optimal number of network states, we used hard and fuzzy clustering algorithms and the Dunn's index as a validity function (Bezdek & Pal, 1998; Carrillo‐Reid et al., 2008, 2009; Sasaki et al., 2007). (c) To visualize clusters of data points representing similar population vectors (i.e., network states) following activity sequences or closed cycles we projected vectors into a two‐dimensional space (Jaidar et al., 2010; Liu, Khalil, & Oweiss, 2011; Sasaki et al., 2007; Schreiber, Fellous, Whitmer, Tiesinga, & Sejnowski, 2003). (d) The position of all cells involved in each state was plotted onto the original image of the slice containing the defined neuronal contours (see previous paragraph).

### 2.8 Statistical analyses

Experimental and control sample size was chosen based on previous literature (Lee et al., 2016). The Shapiro–Wilk test was applied to all original data to assess normality in data distribution. Data withdrawn from a normal distribution were analyzed with the parametric statistical significance test one‐way ANOVA followed by post hoc Tukey honestly significant difference (HSD). When necessary, a paired‐*t* test was also used. Results were expressed as *M* = mean, *SD* = standard deviation and *n* = number of cases; statistical probability values were expressed in the results and figure legends. A simple randomization (i.e., heads‐continuous; tail‐pulses) was applied when the same slices were activated with pulsed or continuous light.

### 2.9 Histology

Following a brief rinse in phosphate buffer 0.01 M, mice were perfused with 4% paraformaldehyde and 14% picric acid; brains were post‐fixed for at least 2 hr and then cryoprotected in a 50/50 mixture of fixative and 20% sucrose in 0.01 M phosphate‐buffered saline (PBS) for at least 24 hr. Sections were cut at 60 μm on a sledge microtome with a freezing stage (Yamato REM‐710 electrofreeze MC‐802A), washed in PBS and incubated in 20% normal goat serum. Primary antibodies to tyrosine hydroxylase (rabbit polyclonal 1:5,000, Enzo Life Sciences) or glial fibrillary acid protein (GFAP, rabbit polyclonal, Dako; 1:500) were incubated overnight at 4°C and stained with secondary goat antirabbit antibodies (Life Technologies; 1:200). At least 2 hr were allowed for binding before rinsing in PBS. Sections were mounted on slides; Vectamount AQ (Vector) or occasionally Santa Cruz mountant with DAPI was used to fix the coverslips. A spinning disk confocal microscope (Olympus BX‐DSU) was used and pictures were taken using Neurolucida software and a Hammatsu (EM‐CCD C91) camera.

## 3 RESULTS

### 3.1 Spontaneous calcium activity in corticostriatal slices of intact or DA‐depleted mice

**Figure 1:**
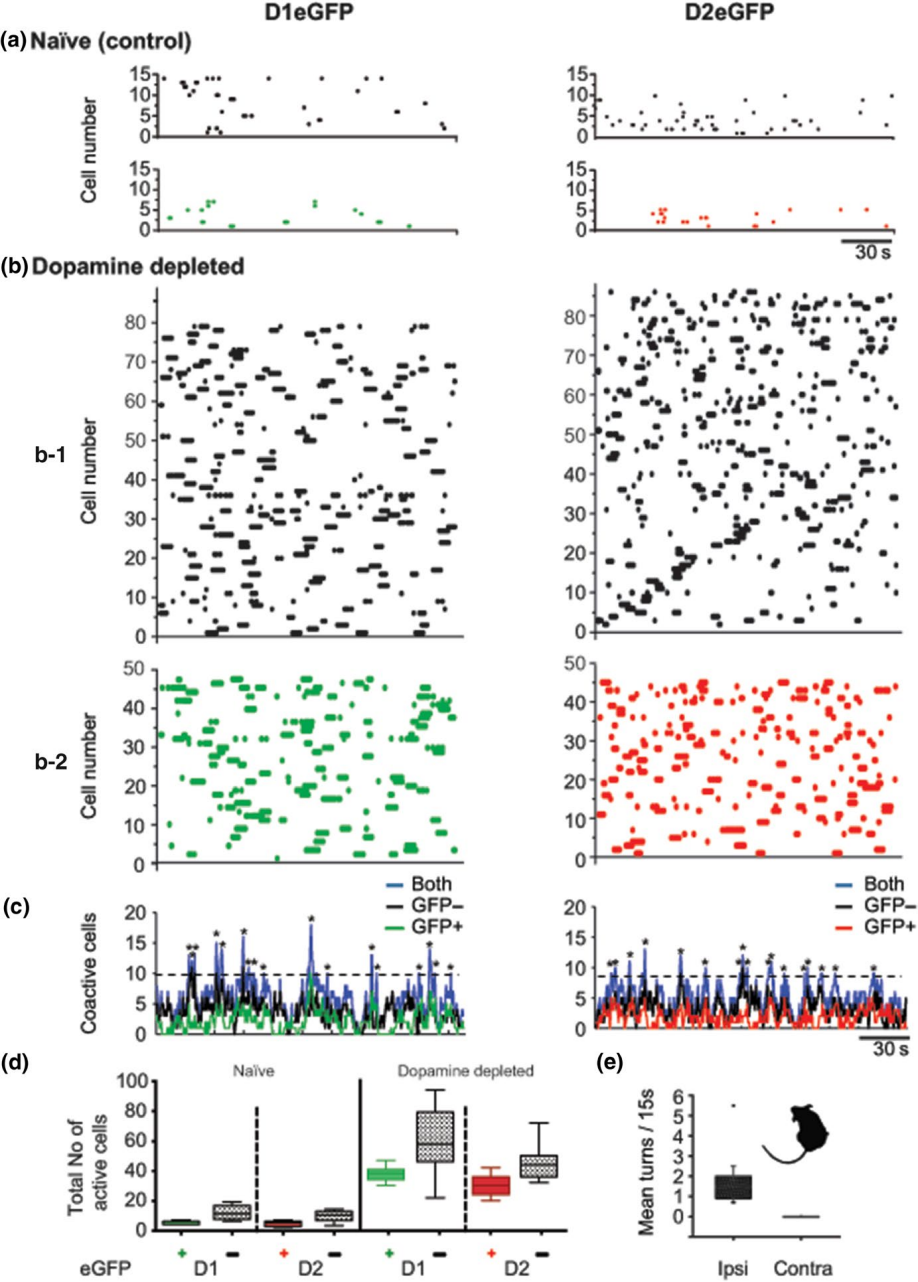
SPNs of the direct or indirect output pathways (D1‐dSPN or D2‐iSPN) contribute to the synchronized network activity after DA depletion. Data are displayed in raster plots where every dot at any given time along the *x* axis represents an active neuron identified by its unique number in the *Y* axis. (a) Representative raster plots of spontaneous striatal activity from D1eGFP (left) and D2 eGFP (right) unlesioned mice. (b) Representative raster plots (top) of D1eGFP (left) and D2eGFP (right) from DA‐depleted animals. (b1) Raster plots of eGPP‐negative neurons, that is, interneurons and the unlabeled SPNs (black dots), (b2) Raster plots of eGPP‐positive neurons of D1 (green) and D2 (red) mice. The increment of neuronal activity after DA depletion compared to controls was statistically significant (one‐way ANOVA (3,28) = 27.12, *p* < 0.0001). (c) Plots of coincidently firing cells (identified by colors: black: eGFP ^−^ negative; green: D1eGFP positive; red: D2eGFPpositive; blue: all recorded neurons, that is, eGFP positive and eGFP negative^−^ together). (d) Box plots of total number of spontaneous active neurons recorded in periods of 180s. (e) Box plot of spontaneous mean rotations recorded on separate experiments. Rotation was toward the side depleted of DA (*M* = 1.77 turns/15 s, *SD* 1.35, *n* = 10 mice). For later comparisons with turns obtained with optogenetic stimulation, turns in DA‐depleted mice were also recorded for 15 s after a 5–10 min period. A contour detection snapshot of a recorded mouse illustrates the deviated posture typical of an ipsilateral DA depletion

The proposed involvement of DA in the generation of striatal neuronal assemblies (Burke, Rotstein, & Alvarez, 2017; Nicola, Surmeier, & Malenka, 2000; O'Donnell, 2003) became testable through calcium imaging that allows simultaneous measurement of many individual neurons (Carrillo‐Reid et al., 2008; Jaidar et al., 2010). With the use of population calcium imaging that allows the indirect measurement of neuronal spiking in striatal networks in vitro (Carrillo‐Reid et al., 2008; Jaidar et al., 2010; Lopez‐Huerta et al., 2013) we characterized the population activity of SPNs in slices obtained from naïve controls and mice depleted of DA for at least 9 days. Examples of simultaneous recordings of fluo‐4 calcium signals and analysis of large numbers of D1eGFP^+^ and D2eGFP^+^ SPNs are shown for naïve and DA‐depleted mice in Figure 1 and Supporting Information Video S1. Spontaneous activity of both D1 and D2 SPNs from non‐lesioned mice slices is characterized by sparse events without significant coactivity episodes (Figure 1a), and following DA depletion, a significant increase in the number of D1 and D2 active SPNs relative to controls is observed (one‐way ANOVA (3,28) = 27.12, *p* < 0.0001), accompanied by the presence of significant coactivity peaks absent in non‐lesioned controls (Figure 1b). As indicated in the graphs of coactive cells (Figure 1c), we counted calcium fluorescent signals not only on eGFP‐positive neurons (D1‐green dots‐ or D2‐red dots‐) but also in SPNs with negative eGFP expression (black dots) that represent the other half of SPNs (D1 or D2) and the interneurons. Group analyses of the number of active SPNs and the number of calcium events of DA‐depleted and control slices revealed as that slices from DA‐depleted slices showed increases of active SPNs for D1eGFP^+^‐ and D2eGFP^+^‐expressing groups, D1 (green): *M* = 37.7, *SD *= 5.33, *n* = 11 mice, and D2 (red): *M* = 30.18, *SD* = 7.01, *n* = 11 mice, whereas control animals exhibited very few active D1 or D2 neurons for the duration of the measurements (3 min), D1: *M* = 4.8, *SD* = 1.3, *n* = 5 mice and D2: *M* = 4.4, *SD* = 1.94, *n* = 5 mice. The coactivity histograms (Figure 1d) are consistent with an increase in activity in both populations (green and red boxes), and also with significant increases in cell activity and peaks of coactivity in the eGFP‐negative neurons (gray boxes). This is a corroboration that most SPNs and possibly interneurons become active in the absence of DA.

**Figure 2:**
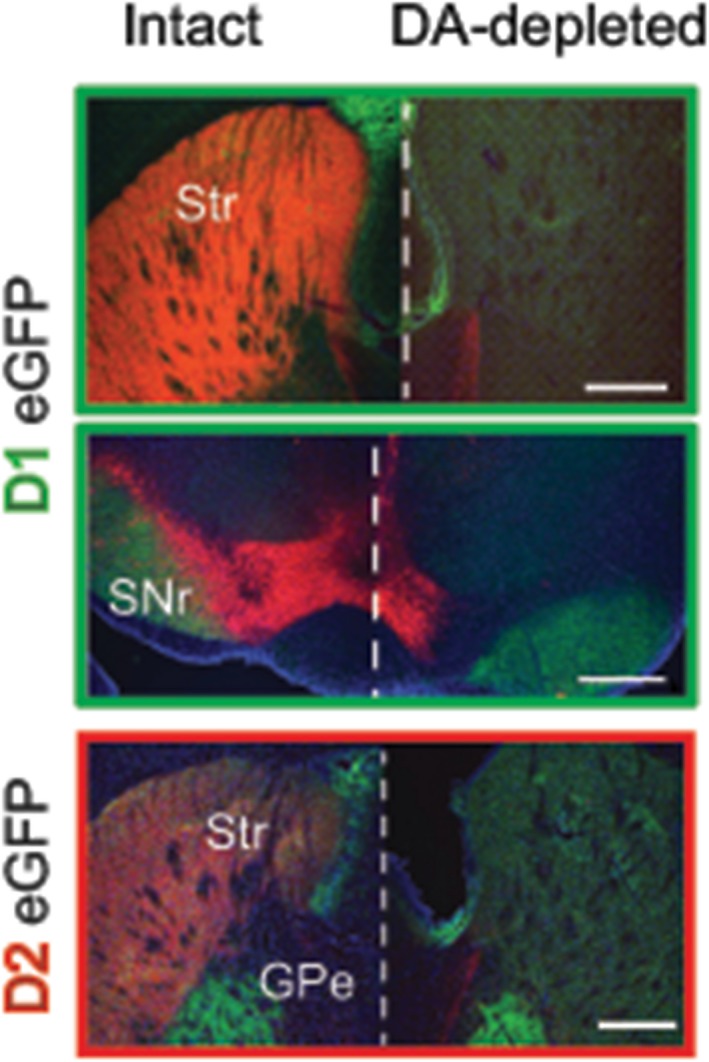
Immunohistochemistry of control and dopamine‐depleted brains. Coronal brain slices of intact and DA‐depleted D1eGFP+ and D2eGFP+ mice illustrate DA neurons and axons by tyrosine hydroxylase (TH) staining (red); positive expression of fluorescent protein (green) and the presence of cell bodies stained with 4,6‐diamidino‐2phenylindole, dihydrochloride (DAPI) (blue). GPe: external globus pallidus; Scale bars: 1 mm; Str: striatum; SNr: substantia nigra pars reticulata

Similar results were obtained from neurons with negative eGFP expression (black dots) (Figure 1b1): a low number of active neurons in the control group (Figure 1a) (D1: *M* = 11.8, *SD* = 4.96, *n* = 5 mice and D2: *M* = 9.6, *SD* = 4.03, *n* = 5 mice) and high number of active neurons in the DA‐depleted slices (Figure 1b) (D1: *M* = 59.6, *SD* = 5.33, *n* = 11 mice and D2: *M* = 30.18, *SD* = 7.01, *n* = 11 mice). The increase in the number of active neurons was statistically significant when compared to controls (Figure 1d). DA depletion was corroborated with immunohistochemistry (Figure 2) and turning behavior (see Section 3). Animals depleted of DA displayed spontaneous turning toward the side of the lesion (*M* = 1.77 turns/15 s, *SD* = 1.35, *n* = 11 mice) (Figure 1d).

**Figure 3:**
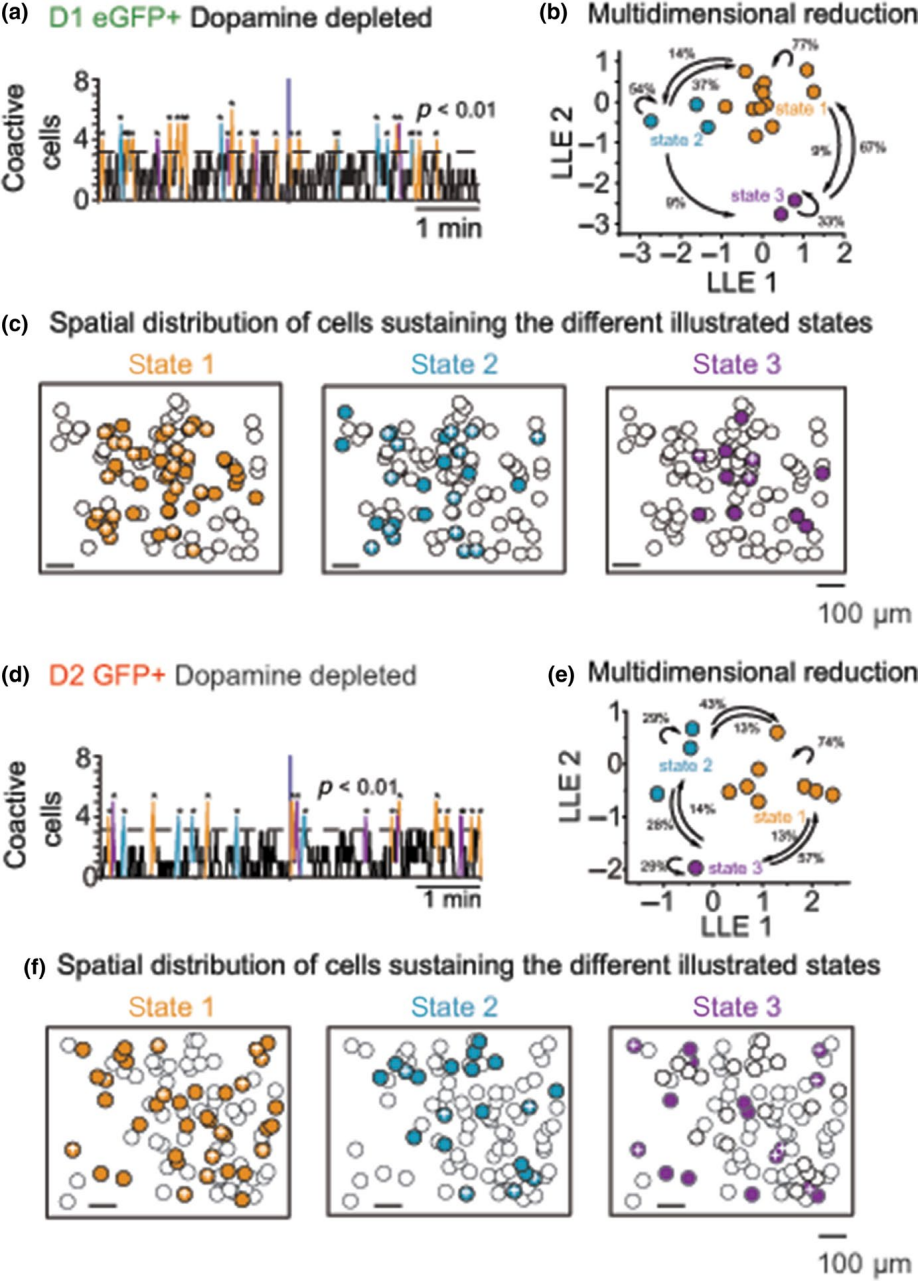
D1 or D2eGFP expressing neurons formed three sets that generated sequential activity states with one predominant set. (a and d) Analyses of coactive cells with significant peaks of synchrony (above the dotted line) also seen in Figure 1c were used for multidimensional reduction (see 4). (b and e). The network states reconstructed from the raster plots show that D1 or D2SPNs generate three sequential activity states (one color per state). The numbers indicate the percentage of transitions between states in the direction of the arrows. The “orange” state clearly dominates. (c and f). Superimposed onto the ROI or map of all observed active neurons, the spatial location of active SPNs can be observed for each one of the states. Colored circles represent active neurons that belong to the same state, circles with no color represent active neurons that do not belong to that particular state, and the plus sign inside the colored circles represents positive eGFPs, that is, represented as red green dots in the raster plots of Figure 1

To continue with the analysis of cell activity and peaks of coactivity as illustrated in Figure 1c, we compared network responses induced by DA depletion (Figure 3) using multidimensional reduction techniques as described (see Section 2.7). A previous description of the striatal microcircuit of unidentified SPNs of rats deprived of DA, was characterized by a dominant network state (Jaidar et al., 2010). Similarly, as previously observed in rats (Jaidar et al., 2010) we observed that D1‐ or D2‐eGFP‐expressing neurons formed three sets that generated sequential activity states. Moreover, as observed in rats (Jaidar et al., 2010), one state that comprised the largest number of active neurons became dominant (refer to Figure 3a,b,d,e orange dots). Importantly, the dominant state was formed by SPNs belonging to both striatal output pathways as illustrated by the presence and absence of the plus sign in active neurons (Figure 3c,f).

### 3.2 Simultaneous photoactivation of direct and indirect pathway SPNs

Consistent with the coordinated synchronous activation of indirect and direct SPNs necessary for the selection, initiation and performance of a particular movement, it has been reported that when the animal is not moving, SPNs remain inactive, but become coactive during movement initiation (Cui et al., 2013; Klaus et al., 2017). With the increase in the number of active cells and peaks of synchrony observed in DA‐depleted tissue, we hypothesized that in the absence of DA, the observed increase in synchronous activation of large sets of striatal neurons could be responsible for the animal's motor impairments, such as deviated posture and persistent spontaneous turning (Costall, Marsden, Naylor, & Pycock, 1976; Dunnett & Iversen, 1982), Figure 1e.

**Figure 4:**
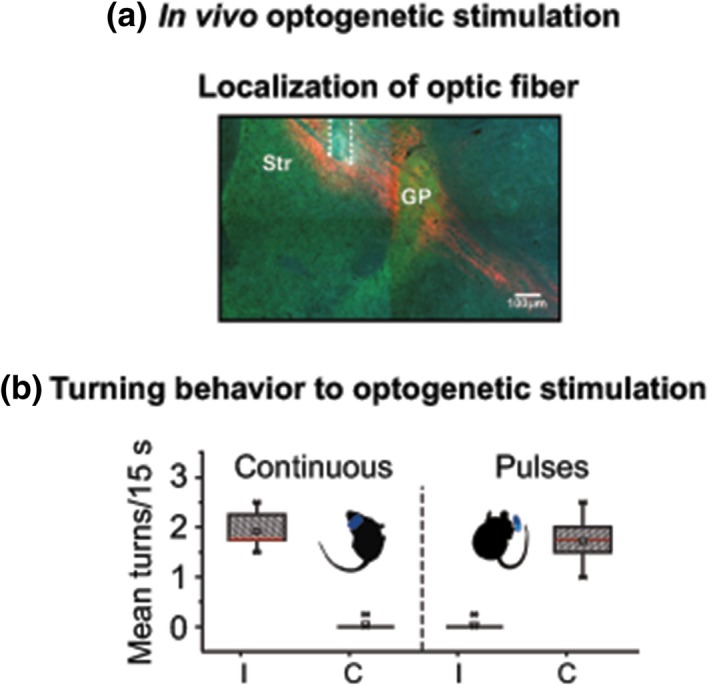
Photostimulation. (a) Photomicrograph illustrating the location of the stimulation optic fiber fixed in the dorsolateral striatum as delineated by dashed white line. (b) Box plots summarizing all behavioral sessions during optostimulation. With continuous light (15 s) (left) animals displayed turning behavior similar to 6‐OHDA treated animals, that is, toward the side stimulated. With stimulation in patterned pulses (14 Hz) animals displayed turning behavior opposite to the stimulated side (right). Behavioral sessions lasted approximately 10–15 min divided as follows: a period of habituation (≈5 min) followed by three sequences repeated 10 times: 1–20 s pre‐stimulation, 2–15 s of light stimulation either delivered continuously or in pulses and 3–30–60 s post‐stimulation. As described in Section 3, a tracking system was used to automatically measure turning. A contour detection snapshot of a recorded mouse illustrates the deviated posture typical of an ipsilateral DA depletion, but in this case in an intact mouse during optogenetic stimulation

To examine the relationship between the increment of neuronal activity and behavior, animals expressing AAV10 ChR2‐mCherry (EF‐1promoter) were used for the unilateral optogenetic activation through an optic probe implanted into the dorsolateral striatum (Figure 4a). We delivered to our behaving animals stimulation at the same intensity (470 nm; 1.0 mW at the tip) for 15 s in two modes as reported by others, for example, a single continuous 15 s pulse (Kravitz et al., 2010) or patterned pulses at 14 Hz (210 pulses of 6 ms each: (Jin, Tecuapetla, & Costa, 2014). When stimulation was delivered in pulses (14 Hz, 6 ms) it induced turning toward the opposite side characteristic of the activation of striatal cell assemblies (Ossowska & Wolfarth, 1995) (*M* 1.73 turns, *SD* 0.99, *n* = 7 mice) (Figure 4b right panel and Supporting Information Video S3). Interestingly, when light was delivered continuously in control animals, behavior changed drastically, mice turned toward the stimulated side (*M* 1.92 turns, *SD* 0.35, *n* = 9; Figure 4b, left panel). This turning behavior is compelling since it is similar to the one observed in DA‐depleted animals (*M* = 1.77 turns, *SD* = 1.35, *n* = 11; Figure 1e). Optogenetically induced turning to the two types of stimulation was statistically significantly different (one‐tail ANOVA *F*(3,28) = 97.14, *p* < 0.001, *n* = 9, 7 continuous vs. patterned pulses), Figure 4b.

Our results demonstrate that in the absence of DA, spontaneous synchronous patterns of SPN activation are observed in both D1 and D2 SPNs, and that when similar large synchronous patterns of activity are induced in both D1 or D2 SPNs by continuous optogenetic stimulation, normal mice turn in circles as do animals with a DA depletion.

### 3.3 Visualization in vitro of striatal dynamics under optogenetic stimulation

**Figure 5:**
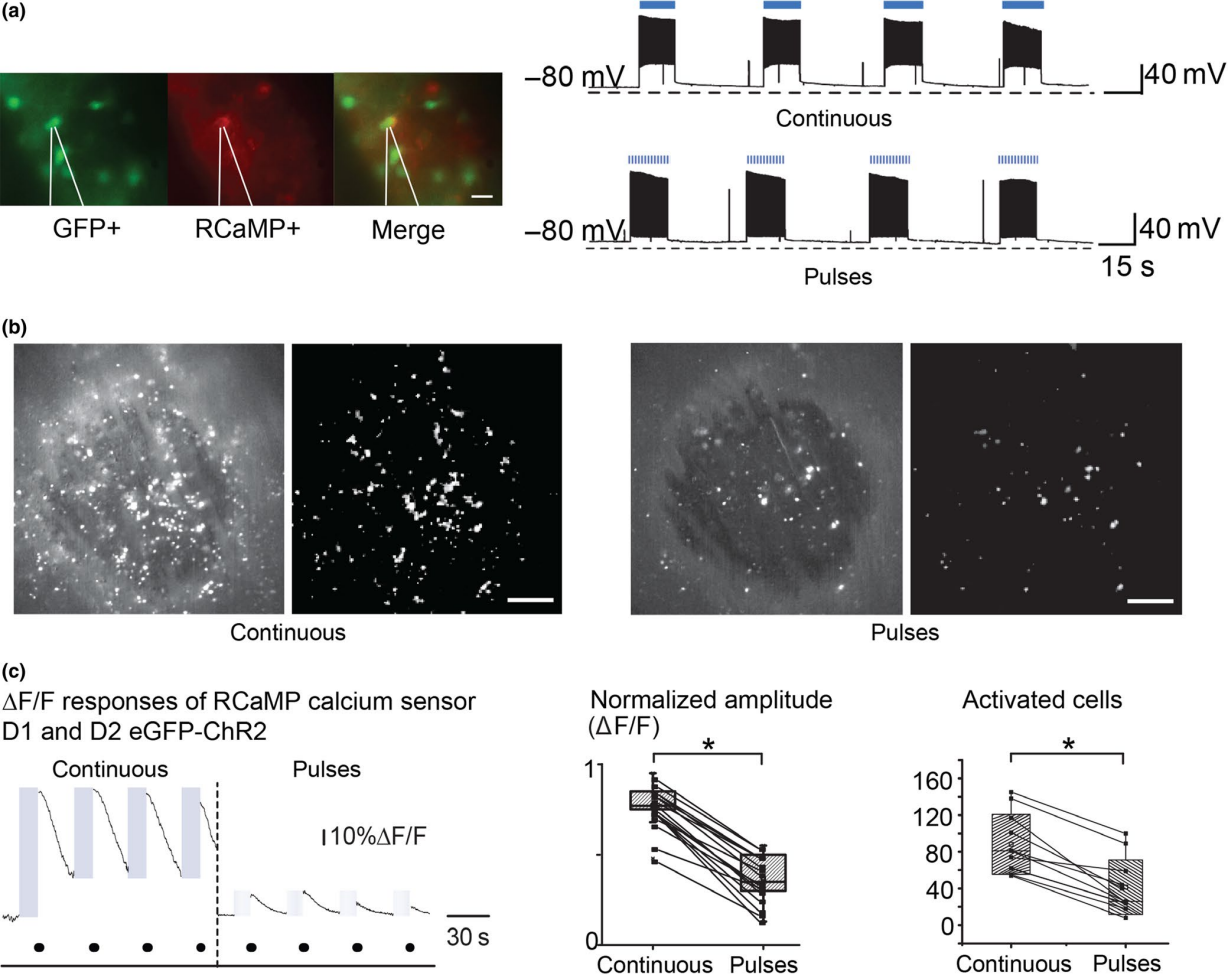
Comparison of responses to in vitro optogenetic stimulation delivered continuously or in patterned pulses. (a) Patch clamp recordings of SPNs expressing ChR2‐RCaMP1 h show normal passive and active membrane properties to 15 s stimulation delivered continuously (top) or in pulses at 14 Hz (bottom). (b) Example of in vitro recorded neuronal response of calcium indicator (RCaMP1 hr) from ChR2‐expressing SPNs with continuous (left) and pulsed (right) light stimulation. (c) Left and middle—Normalized amplitudes of calcium traces from cells activated by continuous stimulation were larger (*M* = 0.77, *SD *= 0.12, *n*17) than those produced by stimulation in patterned pulses (*M* = 0.37, *SD *= 0.13, *n* = 17); paired *t* (16) = 16.22, *p* = 2.33504^−11^). Right— Total number of activated cells from same recorded fields during continuous (*M* = 108, *SD *= 32.63, *n* = 11) and stimulation in patterned pulses (*M* = 61.54, *SD *= 29.65, *n* = 11) paired *t* (20) = 3.5, *p* = 0.00221. Parameters of stimulation were: 15 s of LED of 470 nm blue light stimulation either delivered continuously or in patterned pulses (6 ms pulses at 14 Hz), with intensity at the tip of 1.0 mW

To further visualize how the striatal network elements behaved under optogenetic stimulations we used electrophysiology, calcium imaging, and optogenetics (Figure 5). We combined AAV10 ChR2‐mCherry (EF‐1promoter) with genetically encoded calcium indicator RCaMP1h (hSyn promoter) delivered by AAV1 viral infection. It is relevant to mention that AAV10 ChR2‐mCherry labels the majority of SPNs, but excludes the population of SPN contained in the striosomes (less than 20%) (Lopez‐Huerta et al., 2016).

Neuronal calcium responses were recorded and their amplitude of the responsive cells was normalized by considering the brightest recorded neuronal fluorescence as 100% for each tissue slice. This analysis showed that the normalized calcium response amplitude elicited with continuous stimulation (*M* = 0.77, ∆*F*/*F*,* SD* = 0.1, *n* = 17 slices) was significantly larger than the response observed with stimulation in patterned pulses (*M* = 0.37, ∆*F*/*F*,* SD* = 0.1, *n* = 17 slices; paired *t* (16) = 16.22, *p* = 2.33504^−11^; Figure 5 and Supporting Information Video S2).

Similarly, when the same tissue slices from 11 animals were activated with stimulation delivered at random either continuously or in patterned pulses, the total number of neurons activated by continuous stimulation was significantly larger (*M* = 108 active neurons, *SD* = 32.63, *n* = 11) than the number of neurons activated by patterned pulses (*M* = 61.54 active neurons, *SD* = 29.65, *n* = 11; paired *t* (20) = 3.5, *p* = 0.005, Figure 5d).

Since prolonged electrical stimulation often induces adaptation of SPN discharge (Kita, Kita, & Kitai, 1985) and strong (10–40 mW/mm^2^) prolonged light activation of ChR‐2 can silence instead of activating neurons by depolarization block, particularly interneurons (Herman, Huang, Murphey, Garcia, & Arenkiel, 2014), we first determined in vitro*,* the firing characteristics of our ChR2‐RCaMP1h‐expressing neurons by stimulating with 470 nm (blue light), 1.0 mW at the tip, for 30 s (see Section 3). A total of 14 SPNs (*n* = 7 for each D1 or D2 neurons) responded in similar proportions of activation/adaptation. During depolarization block assessment neurons were exposed to a continuous light pulse for 30 s, the duration of response of activated neurons before undergoing to depolarization block was: D1: *M* = 17.5 s, *SD* = 6.38, *n* = 3 and D2: *M* = 18.25s, *SD* = 11.02, *n* = 4. The other neurons continued firing for the whole exposure time. To our behaving animals stimulation at the same intensity (470 nm; 1.0 mW at the tip) for 15 s in two modes as reported by others, for example, (Jin et al., 2014; Kravitz et al., 2010): a single continuous 15s pulse or patterned pulses at 14 Hz (210 pulses of 6 ms each), all the experimental stimulations were therefore shorter than the average time to cease firing in both groups of cells. Although depolarization block is likely induced in up to half the stimulated cells, the obtained pattern of activation of larger calcium transients was indication that most of the striatal cells were not blocked from firing impulses. The activation was clearly sufficient to induce behavioral changes*,* in vivo, suggesting that the cells excited did influence behavior and so a large recruitment of output neurons is the likeliest explanation of the outcome of the optogenetic stimulation.

**Figure 6:**
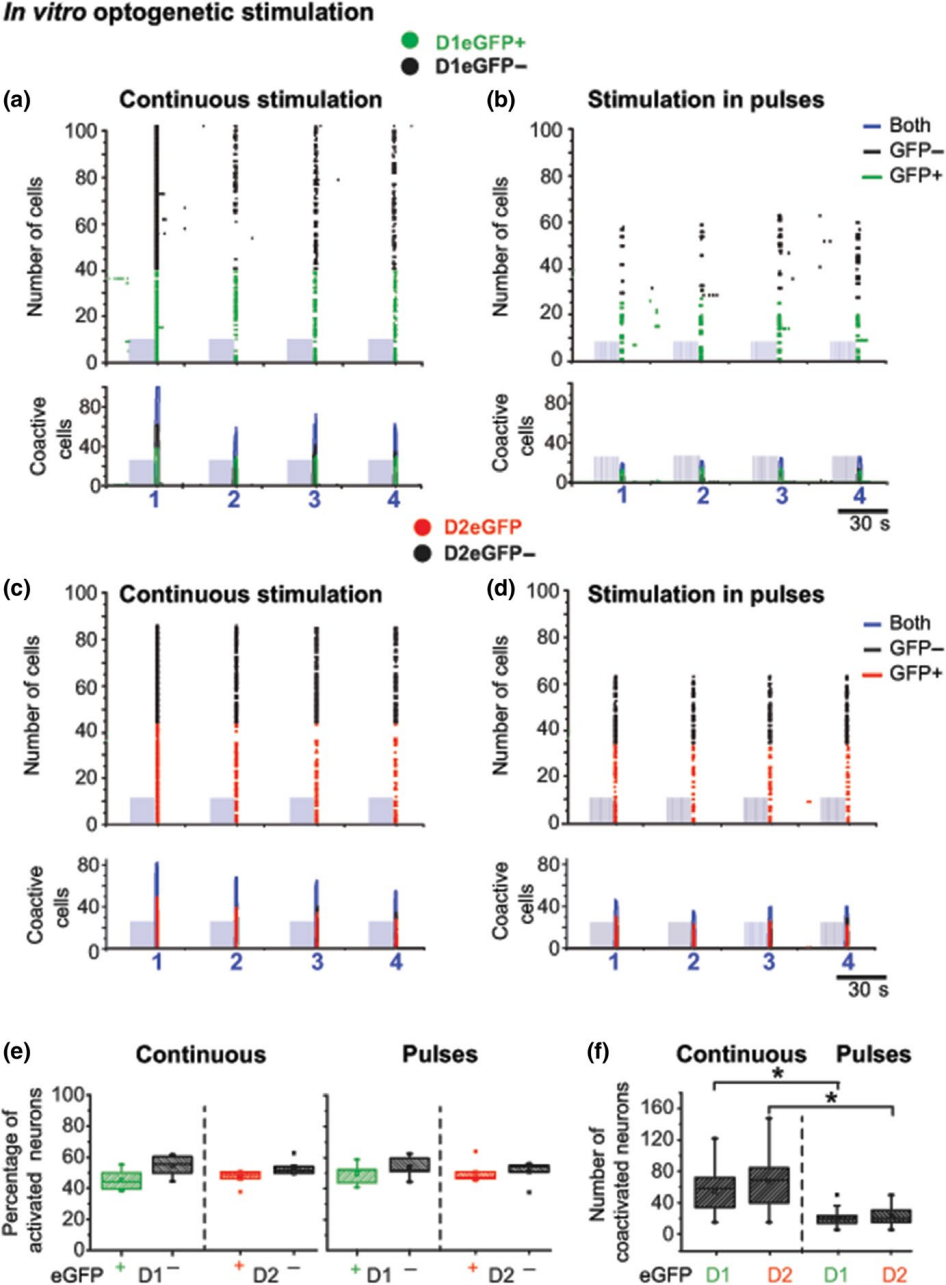
Comparison of SPN activity of synchronous striatal calcium signals induced by continuous and pulsed patterned optogenetic stimulation. Data are represented in sets of two graphs of striatal activity induced by a blue‐light‐emitting diode (470 nm;1.0 mW at the tip) delivered continuously for 15 s or in patterned pulses (14 Hz/15 s). Top traces: illustrate representative examples of raster plots of ChR2‐induced SPN calcium activity reported by RCaMP1 hr. Bottom traces: illustrate histograms of number of coactivated neurons from continuous (a, c, and e left) or patterned pulsed stimulations (b, d, and e right). Blue rectangles represent the light stimulus artifact (see Figure 4a); colors representing individual active neurons and coactivity are, black: all eGFP negative; green: D1eGFP ^+^; red D2eGFP ^+^. (e) In spite of a differential neuronal activation induced by stimulation delivered continuously or in patterned pulses, the percentage of activated SPNs within groups of positive or negative D1 or D2eGFP neurons remains around 50%, as expected. No preferential neuronal activation was observed. (f) Total number of coactivated neurons from both D1 or D2 neurons with positive eGFP expression. Note that both cell types showed higher synchronization to optogenetic stimulation delivered continuously than in patterned pulses (one‐way ANOVA *F*(3,92) = 43.56, *p* ≤ 0.0001)

We studied striatal responses from both direct and indirect pathway (D1eGFP and D2eGFP mice) from the same slices (Figure 6). Light stimulation induced synchronous activation in similar proportions, in both positive and negative eGFP neurons regardless of stimulation delivered continuously or in patterned pulses (D1–D2: range 47–54 synchronous neurons; one‐way ANOVA *F*(7,36) = 1.2, *p* = 0.32, *n* = 5,6; Figure 5a–e), however, it is important to underline that continuous stimulation activated a larger number of both direct and indirect SPNs, whereas patterned pulsed stimulation recruited a smaller number of neurons (continuous D1–D2: *M* = 54.0, *SD* = 27.8, *n* = 24; *M* = 82.6, *SD* = 28.47, *n* = 24; patterned pulses D1–D2: *M* = 19.79, *SD* = 10.13, *n* = 24; *M* = 26.3, *SD* = 11.43, *n* = 24; one‐way ANOVA *F*(3,92) = 43.56, *p* ≤ 0.0001, *n* = 24; Figure 6a–d,f).

**Figure 7:**
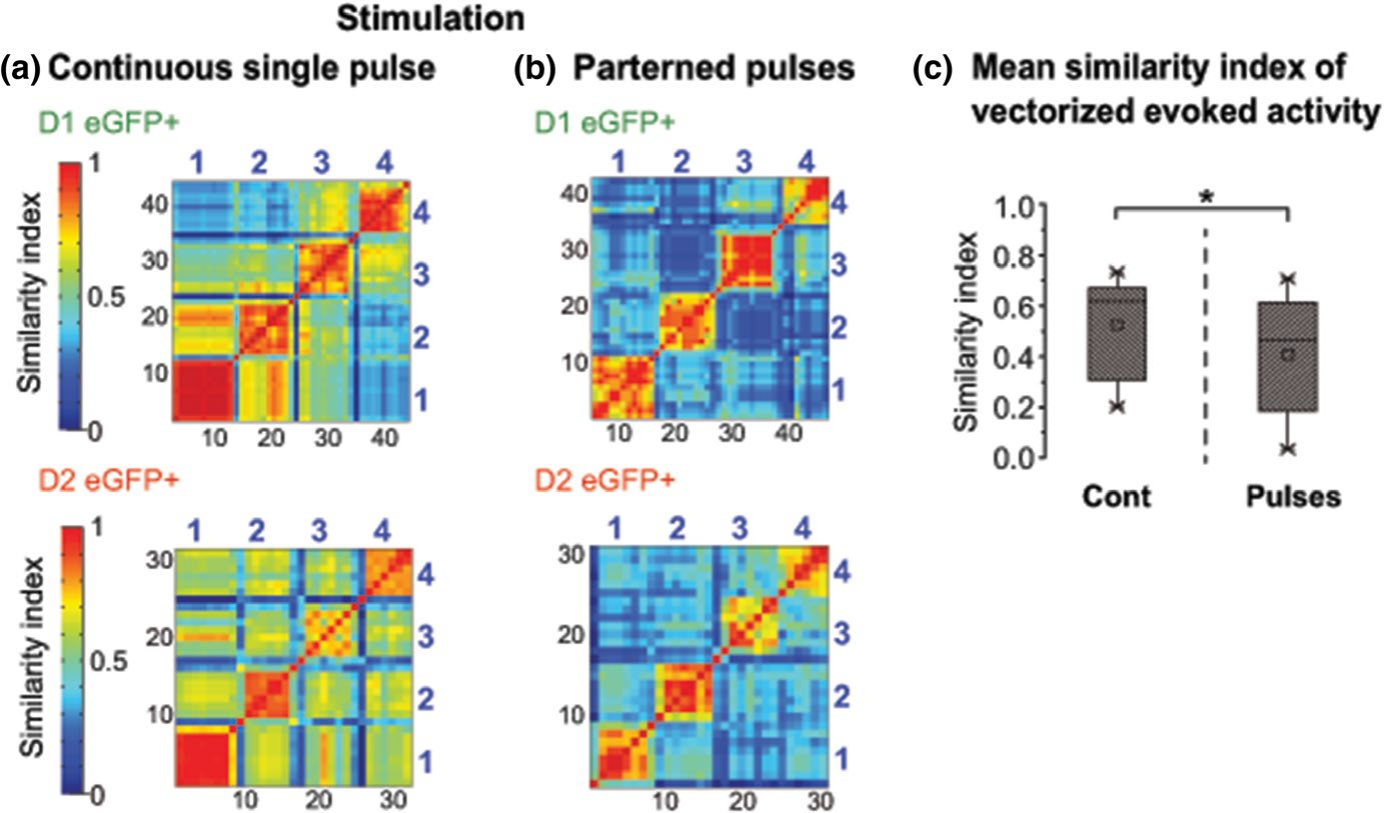
Analysis of network dynamics of D1 and D2 SPNs. The similarity index matrices of the stimulation induced by continuous (a) and stimulation in patterned pulses (b) show that continuous delivery of light activated sets of neurons with higher similarity index across stimulations, than stimulation delivered in patterned pulses. (c) Summary box plots to illustrate the significantly higher mean similarity index of vectorized activity observed with continuous versus patterned stimulation (paired *t* (11) = 4.24, *p* < 0.001, *n* = 12 mice). Black numbers: vector numbers; blue numbers: responses from one of the four stimulations

To visualize the differences in population activity evoked by continuous or patterned optogenetic stimulation we created similarity maps (Carrillo‐Reid, Hernandez‐Lopez, Tapia, Galarraga, & Bargas, 2011; Carrillo‐Reid et al., 2008, 2009; Jaidar et al., 2010) (Figure 7a,b). We found that similarity index between synchronized neurons was significantly higher with continuous stimulation compared to responses following patterned pulses, in other words, continuous stimulation not only activates a larger population of neurons, but it has the tendency of recruit the same neurons across stimuli, whereas patterned pulses recruits fewer and more alternated neurons. Network dynamic between DA‐depleted and pharmacological‐activated striatal cell assemblies, were mimicked by the two forms of light stimulation. That is, pathologically engaged under continuous stimulation and flexible or alternating under patterned stimulation (continuous: *M* = 0.52, *SD* = 0.19 *n* = 12 mice; pulsed: *M* = 0.4, *SD *= 0.23 *n* = 12; paired *t* (11) = 4.24, *p* < 0.001; Figure 7c).

DA modulates short and long‐term SPN function (Gerfen & Surmeier, 2011; Wickens, 2009), modulates its own release (Shen, Flajolet, Greengard, & Surmeier, 2008), pre‐synaptically modulates GABA release at recurrent collateral synapses (Guzmaan et al., 2003; Tecuapetla, Carrillo‐Reid, Bargas, & Galarraga, 2007; Tecuapetla, Koos, Tepper, Kabbani, & Yeckel, 2009), tonically inhibits D2‐SPNs and possibly interneurons too all variables that affect SPN exitability. (Flores‐Barrera, Vizcarra‐Chacon, Tapia, Bargas, & Galarraga, 2010). However, in the unilateral absence of DA, striatal neurons increase their activity and animals display persistent spontaneous ipsilateral turning.

## 4 DISCUSSION

Early studies of striatal cells after DA damage were unanimous in finding the striatal cells more active in anaesthetized animals for example (Arbuthnott, 1974; Hull et al., 1974; Ohye et al., 1970; Schultz, 1982; Schultz & Ungerstedt, 1978). Then came the formulation that made a clear prediction that in PD the direct pathway SPNs should be less active, whereas the indirect pathway SPNs were more active (Albin, Young, & Penney, 1989). This formulation had its origin in changes in genetic activity in the two groups of cells (Gerfen & Young, 1988; Gerfen et al., 1990). The idea was very powerful and led to new therapies including globus pallidus lesions (Laitinen, Bergenheim, & Hariz, 1992), then to subthalamic nucleus deep brain stimulation (Bergman, Wichmann, & DeLong, 1990) and thence to deep brain stimulation (Polack et al., 2007). However, there were difficulties in relating this success to the electrophysiology of individual cells of the two types once they became identifiable by transgenesis. Some laboratories did find differences in the expected direction (Flores‐Barrera, Vizcarra‐Chacon, Bargas, Tapia, & Galarraga, 2011; Shen et al., 2008; Warre et al., 2011) but as the methodology became more sophisticated, the obvious interactions of both cell types with cortical activity and the beta power increase in the electrocorticogram, common in PD, has changed the emphasis from “faster or slower firing” to changes in the intrastriatal dynamics which result from the interactions of the many effects of DA removal on functional relationships within the striatal network (Cui et al., 2013; Klaus et al., 2017; Lindahl & Hellgren Kotaleski, 2016; Parker, Kim, Alberico, Emmons, & Narayanan, 2016; Parker et al., 2018; Perez‐Ortega et al., 2016; da Silva, Tecuapetla, Paixao, & Costa, 2018).

Analyses of many cells at a time have revealed that such neuronal activation is characterized by an increased number of coactive neuronal discharges that involve both types of SPNs (Jaidar, Carrillo‐Reid, & Bargas, 2012; Jaidar et al., 2010). Using calcium imaging in eGFP transgenic animals, we first confirmed the characteristics of SPN activity in the absence of DA, and revealed that both populations of SPNs equally participate in this abnormal microcircuit activity. In our in vitro recordings of spontaneous activity in DA deprived animals, both populations of D1 and D2 SPNs to some extent increased their activity. Therefore, although electrophysiological and anatomical evidence indicates that D2, but not D1 neurons suffer a loss of dendritic spines (Day et al., 2006) but see (Suarez, Solis, Aguado, Lujan, & Moratalla, 2016) and an increase in excitability (Day, Wokosin, Plotkin, Tian, & Surmeier, 2008), in the absence of DA, perhaps the increase in excitability could somewhat compensate the loss of dendritic spines. A recent electrophysiological study in anesthetized rats by Sharott and collaborators claimed that only D2 cells were changed in firing rate but their laborious methodology meant a very small fraction of identified D1 cells in the lesioned animals (*n* = 5 in Figure 3 and *n* = 8 in figure 8) (Sharott, Vinciati, Nakamura, & Magill, 2017). Our method, although in vitro, samples at least 50 cells per slice of both genotypes, the calcium signals are not single action potentials like those of the electrophysiological studies but the likeliest explanation for the transients we measure is a burst of at least three spikes (Carrillo‐Reid et al., 2008). The synchronization of striatal cells is common to both studies, with the Sharott et al. (2017) having correlated the synchronous discharges of D2 cells with beta frequencies in the EEG in their anaesthetized animals; an effect we could not test in vitro. Parker et al. (2018) reported on calcium transients in freely moving animals with a miniature camera and implanted GRIN lens. Although they too see reductions in D1 cell activity, they also see a dislocation of the response of (particularly D2) cells to the initiation of movements after 6‐OHDA injections. During normal movement both D1 and D2 cells are tuned to movements but the rate changes obliterate the movement relations in D2 cells. These different results reflect the within animal control that we lose in our ex vivo models, but we do find a similar disruption of patterned activity in “clusters” of cells that they report.

The effect of bilateral SPN activation using optogenetic techniques has been studied (Parker et al., 2016), but only a few have explored unilateral effects of SPN manipulations (Cui et al., 2013; Jin et al., 2014; Lee et al., 2016). We observed similar results following stimulation in patterned pulses. This suggests that stimulation in patterned pulses is sufficient to stimulate striatal SPNs of either type. Consistently, network analyses revealed that stimulation delivered in patterned pulses (14 Hz/15 s), compared to continuous stimulation, was less efficient in recruiting neurons, but obviously sufficient to induce contralateral turning. The smaller number of coactivated neurons by pulses (14 Hz) induced contralateral turning characteristic of tonic activation of striatal cell assemblies (Carrillo‐Reid et al., 2008; Ossowska & Wolfarth, 1995). Reminiscent of the patterning of responses in striatal cells (independent of output class) observed in the awake mice (Parker et al., 2018). In contrast, from the analyses of network dynamics, it is clear that continuous stimulation induces a significantly higher similarity index compared to patterned stimulation, as also seen in dopamine‐depleted rats (Jaidar et al., 2010).

Therefore, it seems plausible that continuous stimulation induces a dominant recurrent pattern of network activity sufficiently pronounced to induce ipsilateral turning like the spontaneous turning observed in DA‐depleted animals. Such largely synchronous activity patterns would perhaps look “unstructured” if viewed with the small field of view in the in vivo camera, but in our study is obviously a very dispersed concurrent activation. *Pulsed* patterned stimulation could induce a restorative neuronal activity by recruiting fewer coactive neurons. This is a speculative functional relationship obtained from the observed in vitro neuronal network activation by the two stimulation parameters, that remains to be proven with in vivo recordings of neural networks, once technical advances allow such striatal recordings of hundreds of cells, without removal of the overlying cortical tissue.

The reasons for the differences in neuronal recruitment between patterned and continuous stimulation, although interesting, are not the focus of this paper; methodological aspects perhaps involving a ChR2‐activated calcium conductance; reciprocal multisynaptic influences between direct and indirect SPNs and motor cortex (Burke et al., 2017; Freeze, Kravitz, Hammack, Berke, & Kreitzer, 2013; O'Hare et al., 2016; Oldenburg & Sabatini, 2015; Shipp, 2017), intrastriatal influences on SPNs (Planert, Szydlowski, Hjorth, Grillner, & Silberberg, 2010; Taverna, Ilijic, & Surmeier, 2008) and or the participation of interneurons (Damodaran, Evans, & Blackwell, 2014; Dehorter et al., 2009) should be considered in further analyses. Modulation of neuronal assemblies by specific patterns of neuronal stimulation may represent a modified animal model to study the pathogenesis in basal ganglia.

Our purposes were to optogenetically activate selective groups containing both D1‐ and D2‐eGFP‐expressing neurons enough to induce the striatal network activity that presumably supports the spontaneous turning in dopamine‐depleted animals. Therefore, it may be possible that the larger synchronization of the striatal circuit (via continuous stimulation) more closely reproduced the effects of DA depletion. Consequently, the abnormal turning observed in the 6‐hydroxydopamine animal model of PD may result from the pathological striatal synchronous activity that follows DA depletion.

It may be meaningful that to reproduce the pathological state, strong neuronal activation is necessary. It is clear that the stronger continuous stimulation of both D1 and D2 SPNs was required to induce the ipsilateral turning, as it is also the case for the bilateral excitation of D2 SPNs to elicit parkinsonian bradykinesia (Kravitz et al., 2010). A robust inhibition and excitation of different subsets of substantia nigra reticulata neurons has been reported following bilateral striatal optogenetic stimulation of D1 or D2 SPNs, for 1,000 ms but not for 100 ms (Freeze et al., 2013).

Recently, in freely moving animals, it was reported that both SPN populations become active in localized compact groups of synchronized neurons, that alternate their activity over time (Barbera et al., 2016; Klaus et al., 2017; London et al., 2018), these results are similar to previous slice recordings treated with NMDA (Carrillo‐Reid et al., 2008). In contrast, slices from DA‐depleted animals report neuronal activity locked into a dominant network state with reduced alternations between the assemblies (Jaidar et al., 2010; Lopez‐Huerta et al., 2013). Normal striatal motor control requires activation of both direct and indirect SPNs population in a “complementary” and “coordinate” network dynamic (Jin et al., 2014; Klaus et al., 2017; Tecuapetla, Jin, Lima, & Costa, 2016; Vicente, Galvao‐Ferreira, Tecuapetla, & Costa, 2016). Very recent results from a modeling study suggest that for planned movements both output pathways need to be engaged (Bahuguna, Weidel, & Morrison, 2018).

We conclude that the concept of neural networks has provided neuroscientists with an important tool not only to study their properties and capabilities (Blaeser, Connors, & Nurmikko, 2017; Carrillo‐Reid, Lopez‐Huerta, Garcia‐Munoz, Theiss, & Arbuthnott, 2015; Carrillo‐Reid et al., 2009, 2011; Liu et al., 2011), but to activate different functional processes and the related behavior. Fear memories and freezing behavior have been induced by optically stimulating dentate gyrus neurons whose activity during passive avoidance was previously identified (Liu et al., 2012; Ramirez et al., 2013); for review see (Carrillo‐Reid, Yang, Kang Miller, Peterka, & Yuste, 2017).

Here we have used neural network manipulation to demonstrate that: (a) DA depletion caused simultaneous activation of both types of identified D1 and D2 SPNs, (b) the contralateral turning behavior, characteristic of unilateral activated striatal neurons, was induced by patterned pulsed optogenetic stimulation of both types of SPNs, (c) ipsilateral turning behavior, as observed in DA‐depleted animals, was induced in normal animals by continuous optogenetic stimulation that mimics the pathological microcircuit dynamics that accompany this animal model of PD. Our experiments confirm that large, unilateral and synchronous discharges of SPNs can simulate the ipsilateral turning model of PD in intact animals, and suggest that dopamine degeneration causes circling due to a similar large increase in unilateral SPNs discharge. The clinical relevance of this finding is supported by past studies using turning behavior as a model for PD. Resent results implicating glutamate receptors in the hyperexcitability of SPNs in monkeys (Singh et al., 2018) only add to the idea that this striatal pathological activity is close to the symptom development in PD.

## CONFLICT OF INTEREST

There are no conflicts of interest.

## AUTHOR CONTRIBUTIONS

Although with particular predominant and stronger contributions, all authors complied with the criteria for authorship of the European Journal of Neurocience.

## Data Availability

All raw data are stored in the Information Technology section of the Okinawa Institute of Technology Graduate University, Japan and are available upon request to the corresponding author.
