# Supplementary material for: Synchronized activation of striatal direct and indirect pathways underlies the behavior in unilateral dopamine‐depleted mice
Source: Eur J Neurosci. 2019 Jan 30;49(11):1512–28. doi: 10.1111/ejn.14344 (PMC6767564; doi:10.1111/ejn.14344)
Supplement: Supplementary file 4 [file EJN-49-1512-s004.docx]

**Supporting Information**

Supporting video 1. Example of an *in vitro* slice showing striatal spontaneous calcium activity (seen as white spots) obtained from a dopamine depleted animals.

Supporting video 2. Example of an *in vitro* slice showing striatal spontaneous calcium activity induced by four optogenetic stimulati (continuous or pulsed)

Supporting video 3. Turning induced in animals following a 6-OHDA lesion or implanted unilaterally in the dorsolateral striatum with a light probeto deliver unilateral 15 s optogenetic activation either continuously or patterned (6ms pulses at 14 Hz).
