# Supplementary material for: Synchronized activation of striatal direct and indirect pathways underlies the behavior in unilateral dopamine‐depleted mice
Source: Eur J Neurosci. 2019 Jan 30;49(11):1512–28. doi: 10.1111/ejn.14344 (PMC6767564; doi:10.1111/ejn.14344)
Supplement: Supplementary file 5 [file EJN-49-1512-s005.docx]

Synchronized activation of striatal direct and indirect pathways underlies the behavior in unilateral dopamine-depleted

animals

Arbuthnott, Gordon ; Jaidar, Omar; Carrillo.Reid, Luis; Nakano, Yoko; Lopez Huerta, Violeta;

Hernandez Cruz, Arturo; Bargas, Jose; Garcia-Munoz, Marianela

_____________________________________________________________________________________

| Review timeline: | Submission date: 24/07/18  Editorial Decision: Major revision  Revision Received: 30/10/18  Editorial Decision: Minor Revision  Revision Received: 17/12/18  Accepted: 25/12/18 |  |
| --- | --- | --- |
|  |  |  |

Editor: Paul Bolam

Reviewer 1: Anthony West

Reviewer 2: Nicolas Mallet

_____________________________________________________________________________________

1st Editorial Decision	20/09/18

_____________________________________________________________________________________

Synchronized activation of striatal direct and indirect pathways underlies the behavior in unilateral dopamine-depleted animals 

Dear Gordon, 

First of all, apologies for the time it has taken to deal with you manuscript.  It has now been reviewed by external reviewers as well as by the Editorial Team. 

As you can see both reviewers like the paper and consider it an important contribution (as do we) but both raise some issues that will need to be dealt with in a revised version of the paper.   Reviewer 1 simply suggests some more careful proof-reading.   Reviewer 2 however, suggests toning down your assertion that the lack of dopamine leads to a hyperactivity of both populations of MSNs.  He/she also makes the point about the possibility of increased inhibition in the striatum accounting for some of the observations; additional experiments would be good but if not possible then the issue should be clearly discussed.   

We also note the following additional points that will need to be addressed in the revised version. 
- Include the name of the local animal care and use committee.   
- Please check over bibliography missing information. 
- Graphical abstract needs a text. 
- 'At a loss to explain this, we...' Although the charming 'Arbuthnott style', it probably needs changing! 
- Include the total number of animals used.   
- Provide more details of surgery for vector injections and peri-operative care. 
- Anesthetic for the perfusion? 
- I'm not sure, as described, that the opto part would be reproducible; please provide more detail. 
- Details of secondary antibodies and dilution of the GFAP antibody are needed. 
- Please provide precise P values for those greater than 0.001 
- Higher resolution figures will be needed for publication. 

When revising the manuscript, please embolden or underline major changes to the text so they are easily identifiable and please don't leave 'track change' formatting marks in your paper.  Please ensure that you provide a text and a figure file for the Graphical Abstract (as detailed in the instructions below).  When carrying out your revisions please refer to the checklist below and visit the EJN author guidelines at www.ejneuroscience.org 

When finalized, please upload your complete revised manuscript onto the website, as a Word file (.doc, or .docx).  Please also ensure that a complete set of tables and figures is included as separate files, even if these have not changed from the originals.  At this stage it is necessary to provide high resolution figures.  Please see important instructions below. 

Please go into https://mc.manuscriptcentral.com/ejn - Author Centre - manuscripts with decisions where you will find a 'create a revision' link under 'actions'. We ask that you please indicate the way in which you have responded to the points raised by the Editors and Reviewers in a letter. Please upload this response letter as a separate Word (.doc or PDF) file using the file designation "Authors' Response to Reviewers" when uploading your manuscript files.  Please DO NOT submit your revised manuscript as a new one.  Also, please note that only the  Author who submitted the original version of the manuscript should submit a revised version. 

If you are able to respond fully to the points raised, we would be pleased to receive a revision of your paper within 12 weeks. 

Thank you for submitting your work to EJN. 

Best wishes, 

Paul & John 
co-Editors in Chief, EJN 


Reviews: 

Reviewer: 1 

Comments to the Author 
The authors recorded neuronal activity using fluorescent calcium indicators in D1 or D2 eGFP-expressing mice, and found that following dopamine depletion, both striatonigral and striatopallidal output neurons are involved in the large increase in striatal neuronal activity which was patterned and associated with specific ensembles of striatal output neurons.  Additional experiments were performed using optogenetic control in naïve rats which supported the findings that dominant networks of striatal neurons in both projection neuron subtypes are activated following DA loss and implicated in the circling behavior associated with the unilateral DA depletion model of PD.  The authors speculate that the unexpected increase in D1 neuronal activity may be related to changes in interneuron activity which is highly plausible.  These results are also in line with recent studies in NHP by Stella Papa’s group and studies in parkinsonian rodents and represent an important contribution to the field.  I have no significant criticisms but believe the manuscript could benefit from additional proof reading. 

Reviewer: 2 

Comments to the Author 
This paper by Jaidar et al characterizes the changes in neuronal activity (as measured through calcium signal in vitro) induced by the lack of dopamine in the striatum. The authors rely on transgenic mice (i.e. D1 or D2-EYFP) to identify the specific change in D1 or D2 striatal neurons. They found that both population of striatal neurons increase activity after dopamine loss. In addition, the authors conduct optogenetic experiments in behaving animals to figure out the output of different striatal activation regime (i.e. patterned vs. continuous) on the animal’s circling behavior. The results are intriguing: pattern light stimulation induces contralateral rotations whereas continuous light stimulation induces ipsilateral rotations similar to the ones observed in DA-depleted rats. The authors’ main conclusion is that the ipsilateral circling rotation observed in DA-lesioned rats can be a consequence of striatal hyperactivity following DA loss. Overall the paper is interesting but has important issues that need to be addressed: 

1/ The idea that the lack of dopamine leads to a hyperactivity of both striatal population is far from being established in the field. Here, the document contains many statements such as: page 2 line 51 ‘…destruction of striatal DA input is accompanied by an increase in activity of striatal spiny projection neuron (SPN) activity…’ and page 12 line 401‘It is an undisputed fact that in the unilateral absence of DA, striatal neurons increase their activity...’ that just ignore the mainstream idea. This is perhaps intentional since the authors’ main finding is that:’DA depletion caused simultaneous activation of both types of identified D1 and D2 SPNs’ but I found it very confusing to not discuss this point further. Also, since the authors' findings are different from the ones obtain by Parker et al., 2018 using in vivo calcium imaging, what are the possible factors leading to such differences? 

2/ Another important finding from the authors using optogenetic behavioral experiments is that: (page 13 line 443) ‘… continuous stimulation induces a dominant recurrent pattern of network activity sufficiently pronounced to induce ipsilateral turning like the spontaneous turning observed in dopamine depleted animals’. Considering the strong depolarization block effect observed by the authors using continuous light stimulation protocol, it seems important here to further test if this ipsilateral circling behavior is not caused by increase inhibition within striatal circuits. It would be nice to perform additional optogenetic inhibitory experiments and quantify the net effect of such inhibition on the circling behavior. 

3/ It seems important here to increase the n numbers of the depolarization block experiments in order to better understand how optogenetic stimulation translate into neuronal activity. Also, the part describing the depolarization block experiments is rather brief; It would be nice to add further technical information (i.e. did the authors record the electrical activity of neurons in vitro?). 

------------------------ ____________________________________________________________________________________

Authors’ Response	30/10/18

_____________________________________________________________________________________

Manuscript Number: EJN-2018-07-25793

**Synchronized activation of striatal direct and indirect pathways underlies the behavior in unilateral dopamine-depleted animals**

*Notes from EJN*

For revised manuscripts:

- Changes to the text should be emboldened or underlined in the main document
- Lines should NOT be numbered and do not use ‘track changes’
- Minimize use of abbreviations
- Submit a **word text file** (saved as a .doc or .docx), tables (editable text) and high resolution figures separately: TIFF files of 300 d.p.i. for color/half tone and 800 d.p.i. for line work; or EPS files. All figures and tables must be numbered
- Files should appear in the following order: Reply to Reviewers, Main Text Document (i.e., Title Page, Abstract, Introduction, Materials and methods, Results, Discussion, Acknowledgements, Abbreviations, References), Tables, Figure Legends, Figures, Graphical Abstract Text, Graphical Abstract Figure, Color Agreement Form).
- Supporting information: merge into a single *PDF* all supporting information, including supp. figure legends.
- Media files: video files that should be uploaded with the file designation “Media files”.

**From the editors:**

| Observation | √=done |
| --- | --- |
| Include the name of the local animal care and use committee | √ |
| Check over bibliography missing information |  |
| Graphical abstract needs a text, 2-3 sentences (max 500 characters with space) highlighting the major findings. Please upload the text as a Word document file with the file designation "Graphical Abstract Text".  For the figure, please upload a figure file with the file designation "Graphical Abstract Figure.” The figure should be representative of your manuscript and should contain no caption, heading or outline. | √ |
| 'At a loss to explain this, we...' needs changing | √ |
| Include the total number of animals used | √ |
| Provide more details of surgery for vector injections and peri-operative care. | √ |
| Indicate anesthetic for the perfusion | √ |
| Please provide more detail of the optogenetics method | √ |
| Details of secondary antibodies and dilution of the GFAP antibody are needed | √ |
| Please provide precise P values for those greater than 0.001 | √ |
| Higher resolution figures will be needed for publication | √ |

**Authors' Responses to Reviewers**

**Reviewer 1**

The authors recorded neuronal activity using fluorescent calcium indicators in D1 or D2 eGFP-expressing mice, and found that following dopamine depletion, both striatonigral and striatopallidal output neurons are involved in the large increase in striatal neuronal activity which was patterned and associated with specific ensembles of striatal output neurons.  Additional experiments were performed using optogenetic control in naïve mice which supported the findings that dominant networks of striatal neurons in both projection neuron subtypes are activated following DA loss and implicated in the circling behavior associated with the unilateral DA depletion model of PD.  The authors speculate that the unexpected increase in D1 neuronal activity may be related to changes in interneuron activity which is highly plausible.  These results are also in line with recent studies in NHP by Stella Papa’s group and studies in parkinsonian rodents and represent an important contribution to the field.  I have no significant criticisms but believe the manuscript could benefit from additional proof reading.

**Observation.**- Some more careful proof-reading.

**Reply**

More careful proof-reading has been done to the whole document.

**Reviewer 2**

This paper by Jaidar et al characterizes the changes in neuronal activity (as measured through calcium signal in vitro) induced by the lack of dopamine in the striatum. The authors rely on transgenic mice (i.e. D1 or D2-EYFP) to identify the specific change in D1 or D2 striatal neurons. They found that both population of striatal neurons increase activity after dopamine loss. In addition, the authors conduct optogenetic experiments in behaving animals to figure out the output of different striatal activation regime (i.e. patterned vs. continuous) on the animal’s circling behavior. The results are intriguing: pattern light stimulation induces contralateral rotations whereas continuous light stimulation induces ipsilateral rotations similar to the ones observed in DA-depleted rats. The authors’ main conclusion is that the ipsilateral circling rotation observed in DA-lesioned rats can be a consequence of striatal hyperactivity following DA loss. Overall the paper is interesting but has important issues that need to be addressed:

**Observation.-** Tone down your assertion that the lack of dopamine leads to a hyperactivity of both populations of MSNs.

**Reply.-**

A new paragraph was added at the beginning of the discussion (page 17):

**Observation.-** Increased inhibition in the striatum is possible to account for some of the observations. The issue should be clearly discussed if additional experiments are not possible.

**Reply.-**

While it is true that the result of the dopamine depletion is an increase in the activity of the inhibitory cells in the striatum, I am uncertain what the reviewer is referring to. The inhibitory connections between striatal cells are known to be removed by dopamine depletion e.g., (Taverna *et al.*, 2008; Lopez-Huerta *et al.*, 2013), but that is not an increase in inhibition in striatum, it is a decrease. The only cells that might increase inhibition would be the fast spiking interneurons which are thought to survive the dopamine depletion. They are thought to affect the differential activation of the two output pathways – and we see the opposite, a co-activation.

We have known that the destruction of the striatal cells themselves leads to contralateral circling behavior since before the advent of 6-hydroxydopamine (Anden *et al* 1966), so we expect the same would happen if we inhibited the cells optogenetically, but we did not try that. As we say in the discussion there are many other things to explore to understand what is happening with these non-specific activations, but we were emphasizing only that the effects of dopamine loss can be imitated by optogenetic action of a certain kind, and that the pathological behavior of the cells we see, leads to similar behavior in the animals as the loss of dopamine does.

**Question #1.-** The idea that the lack of dopamine leads to a hyperactivity of both striatal population is far from being established in the field.

Here, the document contains many statements such as:

**page 2 line 51** ‘…destruction of striatal DA input is accompanied by an increase in activity of striatal spiny projection neuron (SPN) activity…’

**Reply.-**

We modified the last sentence of the first paragraph of the Introduction page 3.

**page 12 line 401** ‘It is an undisputed fact that in the unilateral absence of DA, striatal neurons increase their activity...’ that just ignore the mainstream idea. This is perhaps intentional since the authors’ main finding is that: ’DA depletion caused simultaneous activation of both types of identified D1 and D2 SPNs’ but I found it very confusing to not discuss this point further.

**Reply.-**

The first sentence of the previous Discussion has become a new paragraph of 19 lines.

Also, since the authors' findings are different from the ones obtain by **Parker et al., 2018** using in vivo calcium imaging, what are the possible factors leading to such differences?

**Reply.-**

Although Parker et al 2018 findings differ from our results, the authors concentrate on the changes in calcium imaging *related to movement* and record from the dorsomedial striatum. In our *in vitro* system, we cannot do such a thing. We note in the discussion that the responses to movement are the main differences in the results. Of course, that is an important addition to the debate about the interactions of dopamine with movement but one we cannot join in, from our data. In their discussion, as in ours, we try to de-emphasize the insistence on firing rate changes in favor of a more detailed examination of the dynamics of striatal firing in different circumstances. Neither of us have to deal with the complications of anesthetics, but neither of us have an intact cortex which may well bias the resting firing rate differently in the two studies.

**Question #2.-** Another important finding from the authors using optogenetic behavioral experiments is that:

**page 13 line 443** ‘… continuous stimulation induces a dominant recurrent pattern of network activity sufficiently pronounced to induce ipsilateral turning like the spontaneous turning observed in dopamine depleted animals’.

Considering the strong depolarization block effect observed by the authors using continuous light stimulation protocol, it seems important here to further test if this ipsilateral circling behavior is not caused by increase inhibition within striatal circuits. It would be nice to perform additional optogenetic inhibitory experiments and quantify the net effect of such inhibition on the circling behavior.

It seems important here to increase the n numbers of the depolarization block experiments in order to better understand how optogenetic stimulation translate into neuronal activity.

It seems important here to increase the n numbers of the depolarization block experiments in order to better understand how optogenetic stimulation translate into neuronal activity.

**Reply.-**

In the Results, section *Visualization in vitro of striatal dynamics under optogenetic stimulation* (page 15) we rewrote the end of the first paragraph to clarify this point.

Moreover, I would like to clarify to the reviewer that one of the reasons we don’t consider depolarization block as the main cause of ipsilateral circling behavior is because in our hands most of the depolarization block was observed after 15s of continuous stimulation, and our experiments never exceed that length of stimulus. Congruently, depolarization block is a state where neurons cease to fire action potentials. In *ex-vivo* brain slices the most sensitive calcium sensors are capable to detect an increment of cytoplasmic calcium concentration when the neurons have a few action potentials (Carrillo et al., 2008, Jaidar et al 2010, Parker et al., 2018), RCaMP1h, as many other red sensors, has the disadvantage of not been as sensitive as their green homologues, requiring more action potentials to produce a clear signal over its basal fluorescence (Akerboom, J. et al.(2013) *Front Mol Neurosci*, **6**, 2). Then any subthreshold calcium concentration will be virtually impossible to detect with this sensor. With that said, in our preparation we observed that during continuous stimulation neurons exhibited fluorescent amplitudes that were significantly larger than the ones generated with pulsed stimulation presumably underlying higher number of action potentials. Moreover, we detected calcium signals from a larger number of neurons when the striatum was exposed to continuous stimulation than to pulsed stimulation representing higher and not lower striatal network activity. Therefore, it seems unlikely to us that the optogenetic stimuli have their major action by depolarization block of the neurons.

The stimulation of the SPNs may have been more effective because of an inhibition of fast spiking interneurons (FS) that was greater with the longer pulses, indeed there have been reports of changes in those cells changing the activity of SPNs (Koos, T. & Tepper, J.M. (1999) *Nat Neurosci*, **2**, 467-472 and synchrony in the striatum (Gittis, A.H., et al. (2011) *J Neurosci*, **31**, 15727). There is also evidence that their action is not modified by dopamine loss (Mallet, N. et al. (2006) *J Neurosci*, **26**, 3875) but the different activity in the two pathways is exaggerated by FS activity. Thus, the blockade of FS by our light stimuli would have had a more discriminatory action than just increasing activity in both groups of striatal output cells, which is what we observe.

**Question #3**

Also, the part describing the depolarization block experiments is rather brief; it would be nice to add further technical information (i.e. did the authors record the electrical activity of neurons in vitro?).

**Reply.**

For a better description of those experiments Figure 5 and the Material and methods section: *In vitro recordings of neuronal activity* (page 8) were modified.

| **Other observations from the Journal** | Done= √ |
| --- | --- |
| Does the title page include ‘Running Title’, ‘Keywords’, ‘word counts’? | √ |
| Does it provide a concluding statement highlighting the significance and impact of the results for the neurosciences? | √ |
| Please consult the Author Guidelines (link shown above) that specifies the detailed information that must be described concerning statistical analysis and results and other major issues.  Suppliers of materials should be named and, with the exception of well-known suppliers, such as Sigma, Kodak and Zeiss, their location (city, state, country) included. | √ |
| All sources of funding should be declared in the Acknowledgements. | √ |
| Are all abbreviations/acronyms defined at first mention?  Are all abbreviations/acronyms listed alphabetically and defined at the end? | √  √ |
| Are all cited references listed (in alphabetical order, without numbers) and have you emboldened the Volume number? | √ |
| Are all listed references complete (e.g. all author's and all editor's names and initials, chapter and book titles and chapter page span in edited books)? | Except for references  below |
| Remove white/blank spaces around figures | √ |
| Filenames must not include any spaces or special characters (do NOT use underscores).  Submission files should not exceed 250MB in total | √ |

Bahuguna, J., Weidel, P. & Morrison, A. (2018) Exploring the role of striatal D1 and D2 medium spiny neurons in action selection using a virtual robotic framework. *The European journal of neuroscience*. doi:10.111/ejn.14021

Parker, J.G., Marshall, J.D., Ahanonu, B., Wu, Y.W., Kim, T.H., Grewe, B.F., Zhang, Y., Li, J.Z., Ding, J.B., Ehlers, M.D. & Schnitzer, M.J. (2018) Diametric neural ensemble dynamics in parkinsonian and dyskinetic states. *Nature*. https://doi.org/10.1038/s41586-018-0090-6

2^nd^ Editorial Decision	22/11/18

_____________________________________________________________________________________

Synchronized activation of striatal direct and indirect pathways underlies the behavior in unilateral dopamine-depleted animals 

Dear Gordon, 

Your revised manuscript was re-evaluated by one of the external reviewers as well as by the editorial team and we are pleased to inform you that it will be accepted for publication in EJN after you have dealt with a few minor technical issues.  Once done we will send it off to the Production Editors 

- The figures in the pdf copy that came to me seems to be still of poor quality, please take another look. 
- 'European' is miss-spelt in the author contributions statement. 
- Figure legends need to be included in the main text (at the end). 
- Please upload the graphic abstract text as a separate file. 

Once again, thank you for submitting your work to EJN. 

Best wishes, 

Paul  & John 
co-Editors in Chief, EJN 

Reviews: 

Reviewer: 2 

Comments to the Author 
The authors have addressed/clarified satisfactorily all my previous comments and I don't have any further comments.

_____________________________________________________________________________________

Authors’ Response	17/12/18

_____________________________________________________________________________________

Thank you for the decision. 

Gordon Arbuthnott.
